# Effect of co-culture with *Halomonas mongoliensis* on *Dunaliella salina* growth and phenol degradation

**DOI:** 10.3389/fbioe.2022.1072868

**Published:** 2022-11-21

**Authors:** Jinli Zhang, Bo Huang, Tao Tang

**Affiliations:** CAS Key Lab of Low-Carbon Conversion Science & Engineering, Shanghai Advanced Research Institute, Chinese Academy of Sciences, Shanghai, China

**Keywords:** phenol degradation, *H. mongoliensis*, dunaliella salina, microalgae-bacteria co-culture, microalgae biomass

## Abstract

The discharge of industrial phenol wastewater has caused great harm to the environment. This study aims to construct microalgae and bacteria co-culture system to remove phenol from simulated high-salt phenol wastewater and accumulate microalgae biomass. The degradation of phenol by marine microalgae *Dunaliella salina* (*D. salina*) and phenol-degrading bacteria *Halomonas mongoliensis* (*H. mongoliensis*) was investigated preliminarily, and then the effects of co-culture *H. mongoliensis* and *D. salina* on the degradation of phenol and the growth of *D. salina* were studied. The effects of *D. salina*/*H. mongoliensis* inoculation ratio, light intensity, temperature and pH on the performance of the co-culture system were systematically evaluated and optimized. The optimal conditions for phenol degradation were as follows: a *D. salina*/*H. mongoliensis* inoculation ratio of 2:1, a light intensity of 120 μmol m^−2^ s^−1^, a temperature of 25°C and a pH around 7.5. Under optimal conditions, this co-culture system could completely degrade 400 mg L^−1^ of phenol within 5 days. Correspondingly, the phenol degradation rate of *D. salina* monoculture was only 30.3% ± 1.3% within 5 days. Meanwhile, the maximum biomass concentration of *D. salina* in coculture was 1.7 times compared to the monoculture. This study suggested that this coculture system had great potential for the bioremediation of phenol contaminants and accumulate microalgae biomass.

## 1 Introduction

Phenol and its derivatives are the common organic pollutants discharged by the petroleum refinery, plastic, paper, pulp, pharmaceuticals and coal processing (Zhang et al., 2020), which may cause serious environmental impacts even at low concentrations. Over the past decades, many conventional wastewater treatment techniques have been developed to remove phenol and phenolic derivatives from industrial wastewater, which include solvent extraction, adsorption, coagulation and chemical oxidation (Mohammadi et al., 2015). However, these methods are complex and expensive, and some of them produce harmful by-products which cause secondary pollution and additional costs for treatment (Mohsenpour et al., 2021). Therefore, it is crucial to develop a more economical, eco-friendly and sustainable phenol wastewater treatment technology.

In recent years, the cultivation of microalgae in wastewater has been demonstrated as a potentially energy-efficient and cost-effective method for wastewater treatment (Mohsenpour et al., 2021). In addition, the harvested algal biomass can be made into various biological products, which make microalgal wastewater treatment technology economically feasible (Li et al., 2022). Diverse strains of freshwater microalgae strains have been effectively employed for the degradation of phenol (Surkatti and Al-Zuhair, 2018; Zhang et al., 2020; Wu et al., 2022). Wang et al. (2016) used an adaptive *Chlorella* sp. strain to remove 500–700 mg L^−1^ phenol completely within 7 days under continuous illumination. A filamentous oleaginous microalgae *Tribonema minus* was screened by Cheng et al. (2017) to degrade phenol, and it could degrade phenol efficiently at the initial phenol concentration up to 700 mg L^−1^ Priyadharshini and Bakthavatsalam (2017) investigated the degradation effect of *Chlorella pyrenoidosa* on the phenolic effluent of a coal gasification plant. The results indicated that *Chlorella pyrenoidosa* could degrade more than 90% of 846 mg L^−1^ of total phenolic compounds. However, the industrial phenol wastewater is often accompanied by high salinity, which will inhibit the growth of freshwater microalgae, and then affect the efficiency of phenol removal (Sierra et al., 2019; Mohseni et al., 2021; Sierra et al., 2021). Few marine microalgae have been used to treat phenolic wastewater with high salinity. Das et al. (2016) reported that a novel diatom BD1IITG could only degrade 39.88% and 24% of 50 and 250 mg L^−1^ phenol, respectively, after 8d incubation. Wang et al. (2019) studied the phenol degradation ability of eight marine microalgal strains and found that *I. galbana* MACC/H59 had the best performance, which could completely degrade 100 mg L^−1^ of phenol within 4 days. However, high concentrations of phenol significantly inhibited the growth of *I. galbana*. It can be seen that the marine microalgae showed less phenol degradation efficiency comparing to that of freshwater microalgae in absence of high salinity. Therefore, it is necessary to develop more efficient system for phenol degradation under high salinity conditions.

Many reports have been demonstrated that co-culture of microalgae and bacteria enhanced the phenol degradation and improved the biomass of microalgae. Yi et al. (2020) reported that *Chlorella sp.* monoculture could not degrade 400 mg L^−1^ phenol, and its growth was seriously inhibited. The co-culture of *Chlorella sp.* and *C. necator* could degrade 1,200 mg L^−1^ phenol within 60 h under optimal conditions. Maza-Márquez et al. (2017) developed one biofilm composed of dominant green microalgae and cyanobacteria and bacteria present in olive washing water (OWW). The system was evaluated for its ability to remove toxic compounds from OWW. The removal rate of total phenolic compounds (PCs) was around 90.3% recorded in the photobioreactor at 3 days of hydraulic retention time. Ryu et al. (2017) investigated the feasibility of microalgae and bacteria consortium to treat toxic coke wastewater. The filtered wastewater with 429.0 ± 9.2 mg L^−1^ PCs was incubated with 80%, 60%, 40%, and 20% dilution in batches. After 94 h of cultivation, 100% phenol was removed by the consortium in all the diluted coke wastewater. The co-culture of microalgae and bacteria had shown great potential for phenol wastewater treatment. However, there are only few studies were reported in this research field, especially for phenol wastewater with high salinity.

This study attempted to investigate the possible of co-culture marine microalgae strain *D. salina* and phenol-degrading bacteria *H. mongoliensis* for phenol degradation under high salt conditions. The optimal experimental conditions for phenol degradation were systematically studied, which included *D. salina* microalgae/*H. mongoliensis* inoculation ratio, light intensity, temperature and pH. The degradation performance of this co-culture system applied to a substrate containing 300–600 mg L^−1^ of phenol was investigated under optimal conditions.

## 2 Experiment

### 2.1 Organisms and culture conditions

The marine microalgae *D. salina* was purchased from Shanghai Guangyu Biological Technology Co., LTD., and maintained in petri dishes using BG11 solid medium with 3% NaCl. *D. salina* cells were successively transferred from petri dishes to 250 ml flasks, and then cultivated in 400 ml bubble column photobioreactors with 1% CO_2_ under 120 μmol m^−2^ s^−1^ and 25°C conditions. Phenol-degrading bacteria *H. mongoliensis* (No. 1.7454) was purchased from the China General Microbiological Culture Collection Center and maintained in petri dishes using 2216E solid culture medium. *H. mongoliensis* cells were successively transferred from petri dishes to 250 ml flasks, and then incubated at 30°C on a rotary shaker (150 rpm). The cells of those microorganisms, *D. salina* and *H. mongoliensis*, were harvested during their logarithmic growth phase by centrifugation. All the harvested cells were resuspended into the simulation phenol wastewater with the required biomass density and used in the following experiments.

### 2.2 Phenol degradation performance of *D. salina* and *H. mongoliensis*

The stock solution of phenol (2000 mg L^−1^) was prepared by dissolving the requisite amount of phenol in sterilized BG11 medium with 3% NaCl. The solution of a required concentration of phenol was prepared by diluting the stock solution with the sterilized BG11 medium containing 3% NaCl.

Batch studies on the phenol degradation capability of *D. salina* and *H. mongoliensis* were conducted in a set of 250 ml flasks (working volume 150 ml) with breathable sealing membranes on a rotary shaker (150 rpm) under continuous illumination (120 μmol m^−2^ s^−1^) and a constant temperature of 25°C. The evaluation for *D. salina* was conducted with a starting cell concentration of 0.4 g L^−1^ and a range of phenol concentrations (i.e., 100, 200, 300, 400 and 500 mg L^−1^). One control group without phenol addition was also conducted for comparison. For *H. mongoliensis*, the initial inoculation concentration was set at 0.3 g L^−1^ to test its degradation capability for 400 and 500 mg L^−1^ phenol, respectively. All tests were performed in triplicate.

### 2.3 Phenol degradation performance of co-culture of *D. salina* and *H. mongoliensis*

In order to investigate the effect of *H. mongoliensis* on phenol degradation and the growth of *D. salina*, different amounts of *H. mongoliensis* were co-cultured with *D. salina* at 400 mg L^−1^ phenol. The initial inoculation concentration of *D. salina* was 0.4 g L^−1^. And 0.05, 0.1, 0.15 and 0.2 g L^−1^ of *H. mongoliensis* was added to adjust the microalgae to bacteria ratio to 8:1, 4:1, 8:3 and 2:1, respectively*.* These experiments were conducted in a set of 250 ml flasks (working volume 150 ml) with breathable sealing membranes on a rotary shaker (150 rpm) under continuous illumination (120 μmol m^−2^ s^−1^) and a constant temperature of 25°C. The phenol concentration, biomass concentration of *D. salina*, Fv/Fm and pH were measured daily. All tests were performed in triplicate.

### 2.4 Optimization of operating conditions for *D. salina* and *H. mongoliensis* co-culture

An evaluation of the influence of operating conditions (phenol concentration, pH, light intensity and temperature) on the performance of the *D. salina* and *H. mongoliensis* co-culture was conducted in 250 ml conical flasks (working volume 150 ml) with breathable sealing membranes. The culture parameters at different operating conditions are shown in Table 1. The initial phenol, *D. salina* and *H. mongoliensis* inoculation concentrations were kept at 400 mg L^−1^, 0.4 g L^−1^ and 0.2 g L^−1^, respectively. The initial pH was adjusted using HCl (1 mol L^−1^) or NaOH (1 mol L^−1^) solution to 5.5, 7.5, 9.5 and 11.5, respectively. The light intensities of 120, 240 and 360 μmol m^−2^ s^−1^ were adjusted by changing the distances between the flasks and the LED (maximum light intensity around 2000 μmol m^−2^ s^−1^). The temperature was regulated using a constant temperature water bath and set as 19, 25, 31 and 37°C, respectively.

**TABLE 1:** The culture parameters of different operating conditions*.

Operating conditions	Phenol concentration (mg L^−1^)	pH	Light intensity (µmol m^−2^ s^−1^)	Temperature (°C)
pH	400	5.5, 7.5, 9.5, 11.5	120	25
Light intensity	400	7.5	120, 240, 360	25
Temperature	400	7.5	120	19, 25, 31, 37
Phenol concentration	300, 400, 500, 600	7.5	120	25

* The initial *D. salina* and *H. mongoliensis* inoculation concentrations were kept at 0.4 g L^−1^ and 0.2 g L^−1^ respectively.

The best degradation performance of the co-culture system was investigated with different initial phenol concentrations under optimal conditions. The initial phenol concentrations were adjusted using stock phenol solution to 300, 400, 500 and 600 mg L^−1^, respectively. All tests were performed in triplicate.

### 2.5 Analysis methods

10 ml of sample was daily withdrawn from each flask to measure the residual phenol concentration, cell density, Fv/Fm and pH.

The pH of the sample was measured using a Five Easy pH meter (METTLER TOLEDO) immediately after the sample was harvested. The maximum quantum yield of photosystem Ⅱ was determined using 2 ml of sample. The Fv/Fm value was measured using a fluorescence monitoring system (FMS2, Lufthansa Scientific Instruments Co., Ltd. United Kingdom) after the sample had been stored in dark conditions for 30 min.

1 ml of sample was centrifuged at 6,000 rpm for 10 min to obtain the supernatant. The concentration of residual phenol in the supernatant was measured by the colorimetric assay 4-amino antipyrine method (Zhou et al., 2017). This method involved the use of 4-aminoantipyrine which reacts with phenol at an alkaline pH in presence of potassium ferricyanide to form a red colored antipyrine dye which could be measured spectrophotometrically against a suitable blank at 500 nm. The residual phenol concentrations were calculated by plotting the values against a suitable standard curve (Bera et al., 2017). The removal efficiency of phenol was calculated using Eq. 1:

$$RE\left(\%\right)=\left(C_{i}-C_{t}\right)/C_{i}\times 100$$ (1a)

Where, RE (%) was the removal efficiency of phenol. Ci and Ct were the concentrations of phenol at the initial stage and after the indicated time, respectively.

The biomass concentrations of D. salina and H. mongoliensis in monocultures were determined gravimetrically. Generally, 5 ml of sample was filtered using a pre-dried and pre-weighed cellulose membrane (0.45 µm pore size), washed with deionized water, dried at 105°C until reached the constant weight, cooled in a desiccator and then weighed again. The dry weight of the blank filter was subtracted from that of the loaded filter to obtain the dry weight.

The biomass concentration of D. *salina* in coculture was determined indirectly by measuring the chlorophyll a and b (Chl a + b) concentrations in co-culture according to the method of Russel et al. (2020). Generally, 0.5 ml of sample was centrifuged at 13,400 rpm for 10 min and the supernatant was discarded. Chlorophyll a and b were extracted from the pellets using methanol (1.5 ml) and quantified as described in Pruvost et al. (2011). The concentrations of Chl a + b (mg L-1) were calculated using Eqs 2–4:

$$Chl\;a=\left[-8.0962\times \left({OD}_{652}-{OD}_{750}\right)+16.5169\times \left({OD}_{665}-{OD}_{750}\right)\right]\times 3$$ (2)

$$Chl\;b=\left[27.4405\times \left({OD}_{652}-{OD}_{750}\right)-12.1688\times \left({OD}_{665}-{OD}_{750}\right)\right]\times 3$$ (3)

Chl a+b=Chl a+Chl b (4)

Where Chl *a*, Chl *b and* Chl *a* + *b* are the concentrations of chlorophyll a (mg L^−1^), chlorophyll b (mg L^−1^) and chlorophyll a and b (mg L^−1^), respectively. OD_652_, OD_665_ and OD_750_ are the optical densities of the extraction solution at wavelengths of 652, 665, and 750 nm, respectively.

A standard curve was prepared for measuring biomass concentrations using Chl *a*+*b* concentrations in a series of *D. salina* suspensions. Chl *a* + *b* concentrations and biomass concentrations of *D. salina* were correlated according to the following Eq. 5:

$$Y=0.0175X+0.0128\;\left[R^{2}=0.992\right]$$ (5)

Where, *Y* and *X* are biomass concentrations (g L^−1^) and Chl *a*+*b* concentrations (mg L^−1^) of *D. salina*, respectively.

The biomass concentration of *D. salina* in the co-culture was calculated using Eq. 5 after measuring the concentrations of Chl *a* + *b* in the co-culture.

### 2.6 Statistical analysis

The means and deviations of the three replicates of each treatment and control, at each sampling time, were calculated. Data are presented as mean ± SD. Statistical analysis was carried out using Origin (ver. 9.0) software.

## 3 Results and discussion

### 3.1 The phenol biodegradation capabilities of *D. salina* and *H. mongoliensis*

**FIGURE 1:**
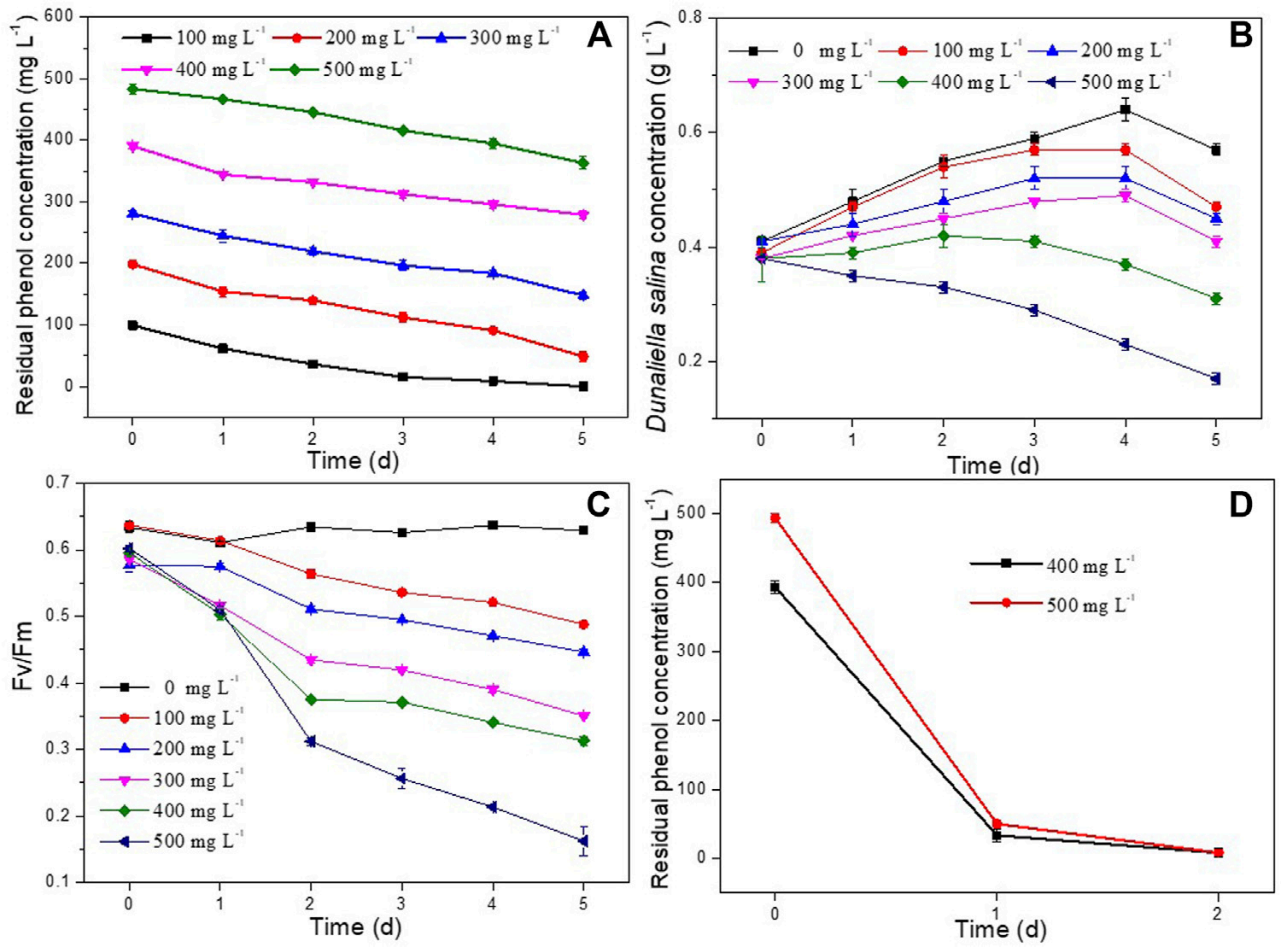
Effect of phenol concentrations on residual phenol concentrations, biomass concentrations and Fv/Fm values of *D. salina* **(A–C)** and *H. mongoliensis* **(D)**.

In order to determine phenol degradation of *D. salina* and *H. mongoliensis,* these two microbes were incubated with different phenol concentrations. *D. salina* was inoculated with 0.4 g L^−1^ biomass concentration and 100–500 mg L^−1^ phenol in the culture medium. As shown in Figure 1A, the residual phenol concentrations decreased with increasing inoculation time across all phenol concentrations. *D. salina* could completely 100 mg L^−1^ phenol within 5 days. The removal efficiency of phenol decreased to 75.5% ± 3.4%, 50.6% ± 6.3%, 30.3% ± 1.3%, 27.3% ± 1.7% with increasing phenol concentrations to 200, 300, 400 and 500 mg L^−1^, respectively. It can be seen that *D. salina* showed poor degradation capacity to high phenol concentrations*.* As shown in Figure 1B, the biomass concentrations of *D. salina* across all phenol concentrations were smaller than that of the control group without phenol*. D. salina* almost could not grow when microalgal cells exposed to 400 and 500 mg L^−1^ phenol concentration, which was consistent with the changes of Fv/Fm values (Figure 1C)*.* The removal efficiency of phenol and growth of *D. salina* were accordance with previous reports (Das et al., 2016; Duan et al., 2017; Wang et al., 2019). Das et al. (2016) reported that the diatom BD1IITG could only degrade 39.88% and 24% of 50 and 250 mg L^−1^ phenol, respectively, after 8d incubation. Wang et al. (2019) investigated the phenol degradation capability of marine microalgae *I. galbana* and found that phenol with concentrations below 100 mg L^−1^ was completely degraded after different residence times of either 2 or 4 days. However, *I. galbana* cells could not grow at phenol concentrations of 225 mg L^−1^. In general, high concentration of phenol exhibits a strong toxicity to microalgae under high salt conditions, which seriously affects the growth of microalgae and even leads to death.

In contrast to *D. salina*, *H. mongoliensis* showed higher phenol tolerance and removal efficiency under high phenol and high salt concentrations. As shown in Figure 1D, *H. mongoliensis* could use phenol as the only carbon and energy source to grow, and completely degrade 400 and 500 mg L^−1^ phenol within 2 days. The results were similar to the phenol degradation capacity of the genus *Halomonas* (García et al., 2004; Ceylan et al., 2011; Haddadi and Shavandi, 2013; Lu et al., 2015). Haddadi and Shavandi (2013) reported that *Halomonas* sp. strain PH2-2 could completely degrade 1,100 mg L^−1^ phenol within 168 h. Lu et al. (2015) Reported that *Halomonas sp.* strain was able to degrade more than 94% of 500 mg L^−1^ phenol over a range of 3%–10% NaCl within 4–5 days. If *H. mongoliensis* are introduced into the phenol degradation system of *D. salina*, it can accelerate the degradation of phenol, thereby reducing the stress of phenol on *D. salina* and promoting the growth of *D. salina*. Therefore, the co-culture of *H. mongoliensis* and *D. salina* might enhance the phenol degradation efficacy and increased the biomass concentration of *D. salina* comparing to *D. salina* monoculture.

### 3.2 The effect of operating conditions on *D. salina* and *H. mongoliensis* co-culture

In order to study the effect of operation conditions on the performance of the co-culture system, operating parameters (inoculation ratio, pH, light intensity and temperature) were systemically investigated. The experimental results are presented and discussed below.

#### 3.2.1 *D. salina* and *H. mongoliensis* inoculation ratio

**FIGURE 2:**
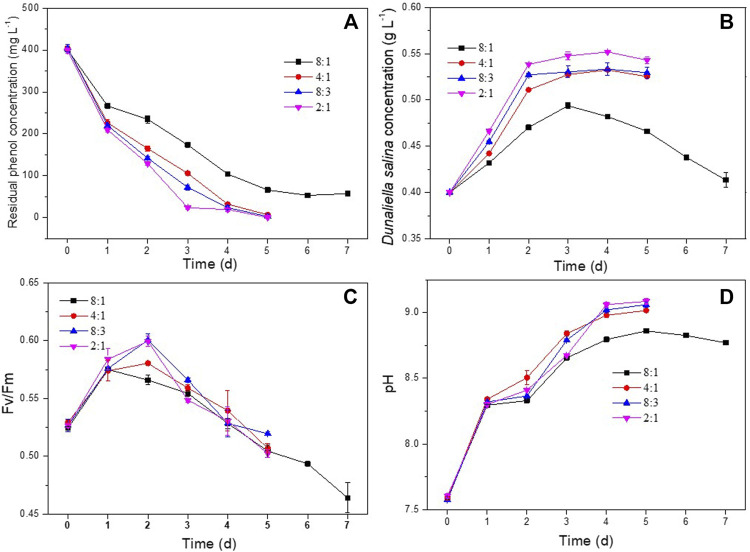
Effect of *D. salina* and *H. mongoliensis* incubation ratio on phenol degradation and the growth of *D. salina*: the residual phenol concentrations **(A)**, biomass concentrations **(B)** and Fv/Fm values **(C)** of *D. salina* and pH of culture medium **(D)** under pH 7.5, 120 μmol m^−2^ s^−1^ and 25°C conditions.

In order to investigate the effect of *H. mongoliensis* addition on phenol degradation and the growth of *D. salinaD. salina*, different amounts of *H. mongoliensis* were co-cultured with *D. salina* at 400 mg L^−1^ phenol. As shown in Figure 2A, the maximum phenol degradation ratio of the co-culture with 8:1 ratio was 85.7% ± 0.9% at 7 days. With increasing the incubation concentration of *H. mongoliensis,* the co-culture with 4:1, 8:3 and 2:1 ratios completely degraded 400 mg L^−1^ phenol within 5 days. These results suggested that phenol removal was likely enhanced by increasing the *H. mongoliensis* inoculation concentration. Comparing to *D. salina* monoculture (Figure 1A), the phenol degradation efficiency of the co-culture system was much higher, which demonstrated the co-culture improved the phenol degradation. In addition, the co-culture also increased the biomass concentrations of *D. salina*. As shown in Figure 2B, the maximum biomass concentration of *D. salina* was 0.55 g L^−1^ for co-culture with 2:1 ratio. However, *D. salina* in monoculture almost could not grow under the same phenol concentration. In this case, the Fv/Fm values of *D. salina* in co-culture were enhanced relative to those of *D. salina* in monoculture (Figure 2C). Hence, the enhancement of photosynthesis increased the biomass concentration of *D. salina* in co-culutre*.* Meanwhile, as shown in Figure 2D, the pH of the culture medium continued to increase with the passage of time due to the enhanced photosynthesis of *D. salina* (Kassim and Meng, 2017). Thus, based on the improvement of phenol degradation and growth of *D. salina*, the feasibility of co-culture *D. salina* and *H. mongoliensis* was demonstrated. These results also suggested the optimal ratio was 2:1 and selected as the following experiments.

#### 3.2.2 pH

**FIGURE 3:**
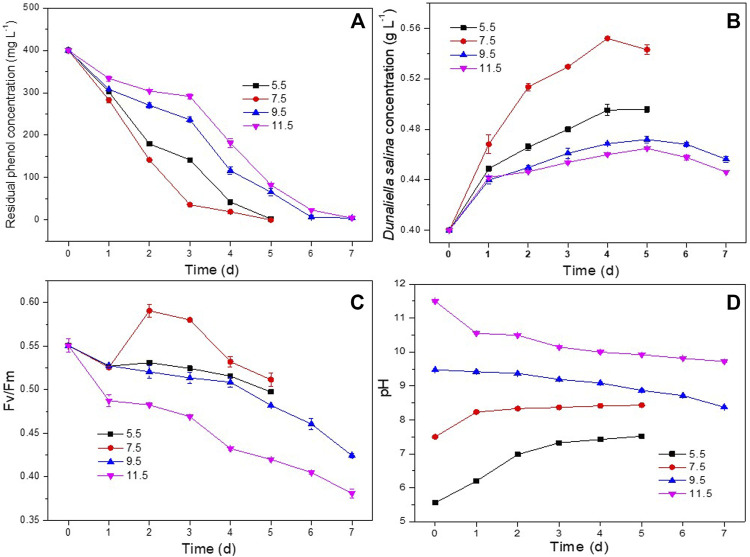
Effect of pH on phenol degradation and the growth of *D. salina*: the residual phenol concentrations **(A)**, biomass concentrations **(B)** and Fv/Fm values **(C)** of *D. salina* and pH of culture medium **(D)** under 0.4 g L^−1^ *D. salina*, 0.2 g L^−1^ *H. mongoliensis*, 120 μmol m^−2^ s^−1^ and 25°C conditions.

The influence of pH on phenol degradation and the growth of *D. salina* was investigated at pH ranging from 5.5 to 11.5. As shown in Figure 3A, the co-culture appeared capable of degrading 400 mg L^−1^ of phenol completely at pH 5.5 and 7.5 within 5 days. Increasing pH to 9.5 and 11.5 resulted in lower phenol degradation efficiency comparing to that at pH 5.5 and 7.5. Correspondingly, as shown in Figures 3B,C, *D. salina* showed higher biomass concentrations and Fv/Fm values at pH 5.5 and 7.5 than these at pH 9.5 and 11.5, and showed the highest biomass concentration at pH 7.5. Similar to the experiments in Section 3.2.1, the pH of the culture medium continued to increase with the passage of time for the co-culture at pH 5.5 and 7.5 due to the photosynthesis of *D. salina*. However, the pH trend reversed for the culture at pH 9.5 and 11.5. (Figure 3D) The decrease of pH could be ascribed to the decrease of the photosynthesis of *D. salina* and the generation of CO_2_ in the process of phenol degradation by *H. mongoliensis* (Borde et al., 2003). In addition, the high alkaline medium was easy to absorb CO_2_ from the air, which led to the decrease of pH value. The results of this experiment suggested that an optimal pH for phenol removal was 7.5.

#### 3.2.3 Light intensity

**FIGURE 4:**
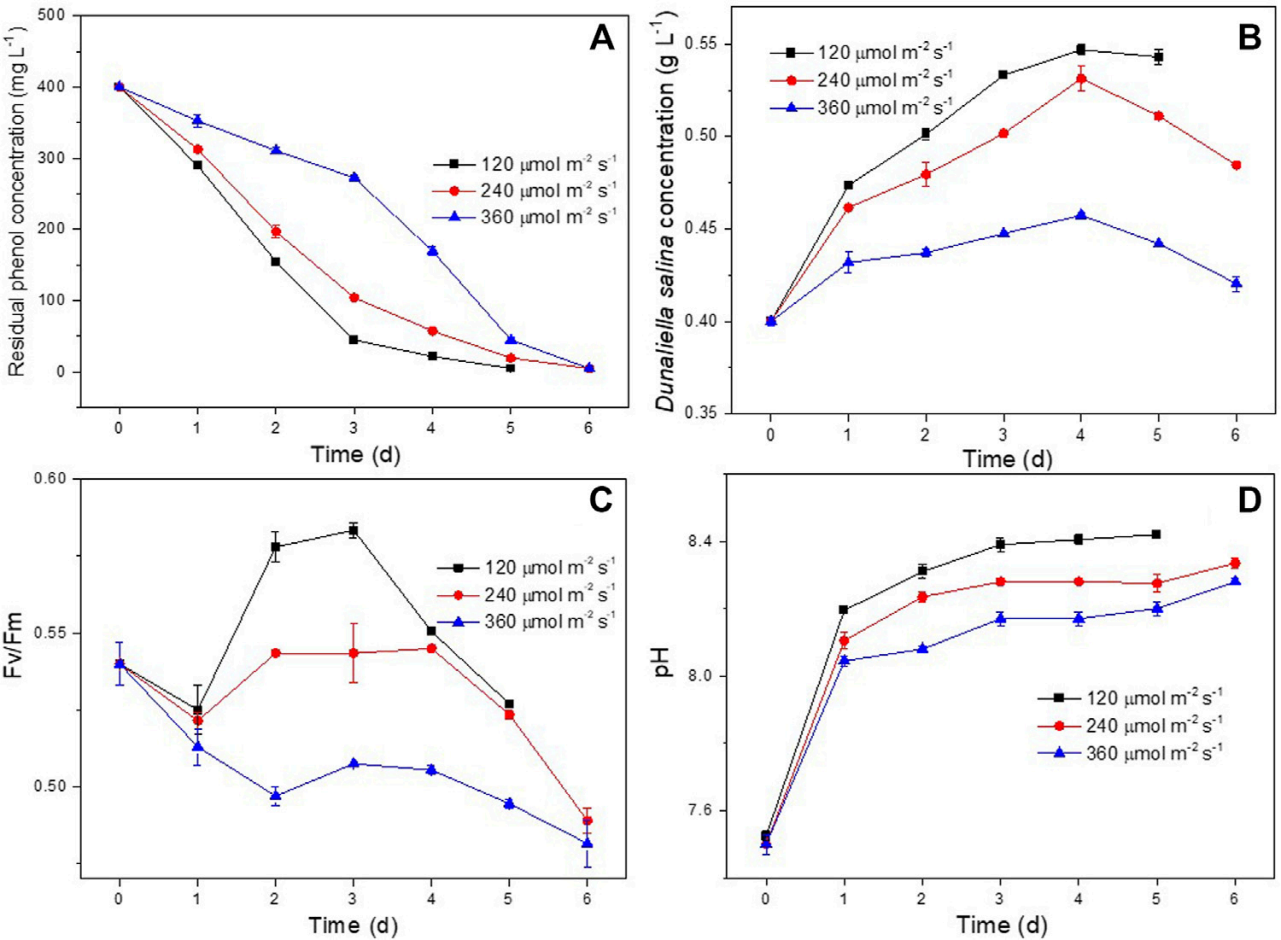
Effect of light intensity on phenol degradation and the growth of *D. salina*: phenol removal efficiency **(A)**, biomass concentrations **(B)** and Fv/Fm values **(C)** of *D. salina* and pH of culture medium under 0.4 g L^−1^ *D. salina*, 0.2 g L^−1^ *H. mongoliensis*, pH 7.5 and 25°C conditions.

The effect of light intensity on phenol degradation and the growth of *D. salina* was studied at light intensities of 120, 240 and 360 μmol m^−2^ s^−1^, respectively. As shown in Figure 4A, the co-culture system could completely degrade 400 mg L^−1^ of phenol completely at 5, 6 and 6 days under 120, 240 and 360 μmol m^−2^ s^−1^, respectively. It showed that high light intensity had negative effect on the phenol degradation. As shown in Figure 4B, the biomass concentrations of *D. salina* cultivated under 360 μmol m^−2^ s^−1^ was much smaller than that of 120 and 240 μmol m^−2^ s^−1^. It suggested that high light intensity inhibit microalgal growth, which can also be confirmed by the obvious decrease of Fv/Fm values (Figure 4C) under 360 μmol m^−2^ s^−1^.The changes of pH under different light intensities (Figure 4D) were similar to that of under different incubation ratios. The results could be ascribed to the damage on photosynthetic organs of microalgal cells caused by photo inhibition resulted from high light intensity. Similar findings have been reported and photo-inhibition was recognized as a reasonable explanation (Chandra et al., 2016; Seepratoomrosh et al., 2016; Seo et al., 2017). However, the phenol also could be completely degraded within 6 days under 360 μmol m^−2^ s^−1^, which could be ascribed to bacterial growth. (Borde et al., 2003; Essam et al., 2013; Maza-Márquez et al., 2017) Basing on the above results, the optimal light intensity for phenol degradation and the growth of *D. salina* was in the range of 120–240 μmol m^−2^ s^−1^.

#### 3.2.4 Temperature

**FIGURE 5:**
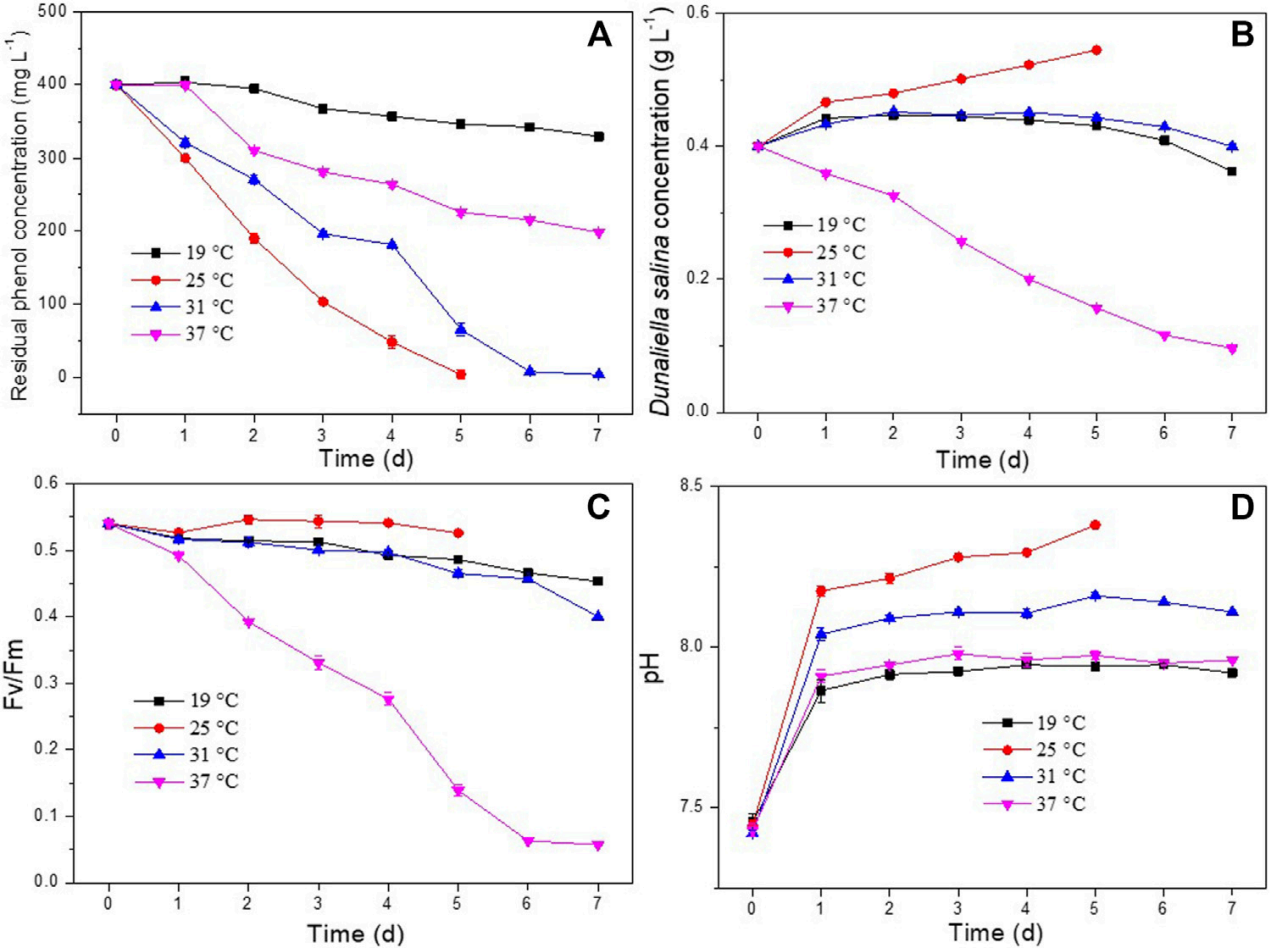
Effect of temperature on phenol degradation and the growth of *D. salina*: phenol removal efficiency **(A)**, biomass concentrations **(B)** and Fv/Fm values **(C)** of *D. salina* and pH of culture medium **(D)** under 0.4 g L^−1^ *D. salina*, 0.2 g L^−1^ *H. mongoliensis*, pH 7.5 and 120 μmol m^−2^ s^−1^ conditions.

Culture medium temperature is an important conditioning factor for microbial growth and metabolic activities (Newsted, 2004; Li et al., 2019; Li et al., 2020). The effect of temperature on phenol removal and the growth of *D. salina* was studied within a range of 19°C and 37°C. As shown in Figure 5A, the co-culture could completely degrade 400 mg L^−1^ of phenol within 5d and 7d at temperatures of 25°C and 31°C, respectively. The phenol removal efficiencies at 19°C and 37°C were much lower than those at 25°C and 31°C. As shown in Figures 5B,C, the biomass concentration and Fv/Fm values of *D. salina* in the co-culture maintained at 25°C were much higher compared to the same factors at 19°C, 31°C, and 37°C. The changes of pH under different temperatures (Figures 5D) were similar to that of under different incubation ratios. The results indicated that an increase or decrease in the temperature outside the optimal range inhibited microalgal growth and activity, and then inhibited phenol degradation. The optimum temperature for microalgae to grow varies with microalgal species and culture medium composition (Zhao and Su, 2014). Pires et al. (2012) reported that temperature higher than 35°C are usually lethal for a number of microalgal species. Generally, a high temperature inhibits the microalgal metabolic behavior and a series of temperature-dependent physicochemical reaction processes such as the benzene ring cleavage in the process of phenol degradation (El-Naas et al., 2009; Zhao and Su, 2014). Whereas, low temperatures affect photosynthesis of microalgal cells by reducing carbon assimilation activity (Khan et al., 2018). Therefore, the optimal temperature for phenol degradation and microalgae growth suggested by this experiment was 25°C.

### 3.3 Phenol concentration

In order to investigate the best phenol degradation performance of *D. salina* and *H. mongoliensis* co-culture, the experiments were carried out for degradation of 300–600 mg L^−1^ phenol under optimal operating conditions (25°C, 120 μmol m^−2^ s^−1^, pH 7.5).

**FIGURE 6:**
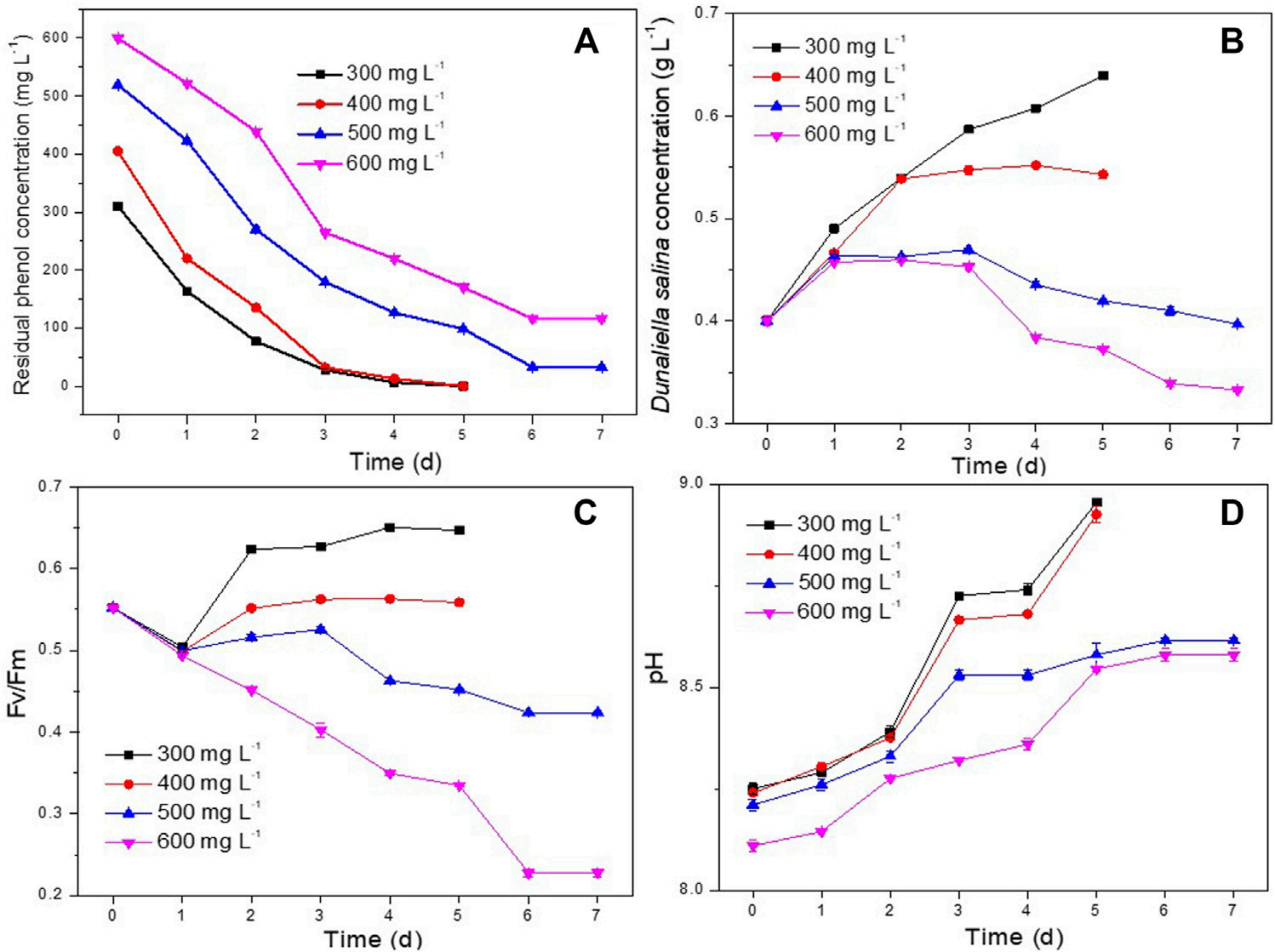
Effect of phenol concentrations on phenol degradation and the growth of *D. salina*: phenol removal efficiency **(A)**, biomass concentrations **(B)** and Fv/Fm values **(C)** of *D. salina* and pH of culture medium **(D)** under 0.4 g L^−1^ *D. salina*, 0.2 g L^−1^ *H. mongoliensis*, pH 7.5, 120 μmol m^−2^ s^−1^ and 25°C condition.

As shown in Figure 6A, both 300 and 400 mg L^−1^ concentrations of phenol were completely degraded within 5 days. The phenol removal efficiencies of *D. salina* monoculture were only 50.6% ± 6.3%, 30.3% ± 1.3% under 300 and 400 mg L^−1^ phenol, respectively. It can be seen that the addition of bacteria enhanced the phenol degradation efficiency. In addition, the maximum biomass concentration of *D. salina* in coculture were 1.5 and 1.7 times compared to the monoculture at 5 days under 300 and 400 mg L^−1^ phenol, respectively. This might be due to the phenol degradation by *H. mongoliensis,* which reduce the stress of phenol to *D. salina* cells. Meanwhile, phenol was degraded to CO_2_ and then enhanced microalgal growth as carbon source (Maza-Márqueza et al., 2013; Ryu et al., 2017).

Under the condition of higher phenol concentration, the degradation rates of 500 mg/L and 600 mg/L phenol in the co-culture system could reach to 93.4% ± 0.3% and 80.6% ± 0.1%, respectively, which is much higher than that of *D. salina* monoculture. However, the biomasses and Fv/Fm values (Figures 6B,C) of *D. salina* in the co-culture system were relatively low. In addition, the pH of the culture medium under 500 and 600 mg L-1 phenol were higher than that of under 300 and 400 mg L-1 phenol (Figures 6D). These results indicated that high concentration of phenol produced great stress on the growth of *D. salina*. However, comparing to previous studies (Das et al., 2016; Wang et al., 2019), the co-culture system of *D. salina* and *H. mongoliensis* in this study showed higher phenol removal rate and efficiency. To the best of the authors’ knowledge, it was the best performance for phenol degradation by microalgae and bacteria microcosm under high salt conditions. Generally, bacteria have higher phenol tolerance and degradation capability than microalgae (Mohammadi et al., 2015; Bhattacharya et al., 2018). It can be seen that the addition of salt-resistant phenol degrading bacteria significantly improved the maximum phenol degradation concentration and phenol degradation efficiency of the co-culture system, and also increased the biomass of microalgae. In order to further increase the efficiency of the system, it is necessary to screen halotolerant microalgal strains with higher phenol tolerance and degradation efficiency to establish an efficient microalgae and bacteria microcosm, and investigate the phenol removal, the growth of microalgae and bacteria and the relationship between them, and discuss the interactions between microalgae and bacteria in the process of high concentration phenol degradation.

## 4 Conclusion

In this study, a co-culture of *D. salina* and *H. mongoliensis* was artificially established for degradation of phenol and accumulate microalgae biomass under high phenol and salt conditions. The efficacy of this technique was demonstrated after conducting experiments to determine optimal operating conditions that included the factors like pH, light intensity, temperature. This co-culture system could completely degrade 400 mg L^−1^ of phenol within 5 days, and the maximum biomass concentration of *D. salina* in coculture was 1.7 times compared to the monoculture. This study suggests that this co-culture has great potential for the bioremediation of phenol contaminants and accumulate microalgae biomass.

## Data Availability

The original contributions presented in the study are included in the article/Supplementary Material, further inquiries can be directed to the corresponding author.
